# A systematic review on leptospirosis in cattle: A European perspective

**DOI:** 10.1016/j.onehlt.2023.100608

**Published:** 2023-07-27

**Authors:** Cynthia Sohm, Janina Steiner, Julia Jöbstl, Thomas Wittek, Clair Firth, Romana Steinparzer, Amélie Desvars-Larrive

**Affiliations:** a VetFarm, Department of Farm Animals and Veterinary Public Health, University of Veterinary Medicine Vienna, Kremesberg 13, 2563 Pottenstein, Austria; b Unit of Veterinary Public Health and Epidemiology, Department of Farm Animals and Veterinary Public Health, University of Veterinary Medicine Vienna, Veterinärplatz 1, 1210 Vienna, Austria; c University Clinic for Ruminants, Department of Farm Animals and Veterinary Public Health, University of Veterinary Medicine Vienna, Veterinärplatz 1, 1210 Vienna, Austria; d Institute for Veterinary Disease Control, Austrian Agency for Health and Food Safety (AGES), Robert Koch-Gasse 17, 2340 Mödling, Austria; e Complexity Science Hub Vienna, Josefstädter Straße 39, 1080 Vienna, Austria

**Keywords:** Cattle, *Leptospira*, Leptospirosis, Europe, Systematic review, Epidemiology

## Abstract

Leptospirosis is a zoonotic disease which is globally distributed and represents a classic One Health issue that demands a comprehensive understanding of the hosts, transmission paths, and risk factors of transmission. Bovine leptospirosis often results in economic losses through its severe impact on reproduction performance while it threatens human health at human-cattle-environment interfaces. However, a clear analysis of the disease characteristics in European cattle is currently lacking.

The objective of this review was to summarise the current knowledge on the epidemiology of bovine leptospirosis in Europe. We conducted a systematic literature review, screening four electronic databases, and filtered articles published between 2001 and 2021, in English, German, and French.

Sixty-two articles were ultimately included in the review. The seroprevalence of leptospirosis in cattle was remarkably variable among studies, probably reflecting local variations but also heterogeneity in the study designs, laboratory methods, and sample sizes. Risk factors positively associated with the disease were diverse, related to local, environmental, and climatic parameters as well as farming practices. The most reported circulating *Leptospira* serogroups in European cattle were Sejroe (58.5%), Australis (41.5%), Grippotyphosa (41.5%), Icterohaemorrhagiae (37.7%), and Pomona (26.4%), which have also been associated with human infections worldwide. Abortion (58.6%) and fertility disorders (24.1%) were the most frequently reported signs of leptospirosis in European cattle and were generally associated with chronic infections.

This work highlights several research gaps, including a lack of harmonisation in diagnostic methods, a lack of large-scale studies, and a lack of molecular investigations. Given that predictions regarding the climatic suitability for leptospirosis in Europe suggest an increase of leptospirosis risk it is important to raise awareness among stakeholders and motivate an integrated One Health approach to the prevention and control of this zoonotic disease in cattle and humans.

## Highlights

• Developing One Health strategy to tackle leptospirosis requires knowledge on hosts**.**
• Sejroe is the most reported serogroup circulating in European cattle (58.5%)**.**
• Abortion is the most frequently reported sign of leptospirosis in European cattle (58.6%)**.**
• Methods for diagnosis of leptospirosis require a pan-European standardisation**.**
• Prevention and control of leptospirosis in cattle requires a One Health approach**.**

## 1 Introduction

Leptospirosis is a neglected zoonotic disease, that is distributed worldwide [1]. The disease is estimated to affect 1.03 million people globally and cause 58,900 deaths annually [2]. Most outbreaks occur in tropical regions, but cases are also reported from temperate areas [3]. The causative bacterial agent, *Leptospira* spp., has been reported in a wide range of mammals worldwide [4]. Transmission occurs primarily through direct or indirect (i.e. via contaminated water or soil) contact with the urine of infected animals [3,5] although venereal transmission is also described [6]. The bacteria can enter the body through the mucous membranes or damaged skin [5]. Following a leptospiraemic phase, the bacteria can colonise various organs, especially the kidneys, from where they are then shed intermittently in the urine [4,5], or the genital tract, where they can be detected in the semen or vaginal discharge [6].

The phylogenetic classification, which organises *Leptospira* species based on DNA relatedness [7], currently acknowledges 68 species [7,8]. It coexists with the historical, serological classification, which recognises more than 300 serovars of *Leptospira*, grouped into serogroups [9] based on the expression of the surface-exposed lipopolysaccharide (LPS).

The gold standard for the detection of leptospiral antibodies is the microscopic agglutination test (MAT), which uses live cultures of *Leptospira* strains that are tested at different dilutions against patient (animal or human) serum [4]. The enzyme-linked immunosorbent assay (ELISA) is also widely used for the serological diagnostic of leptospirosis [5]. Isolation of the bacteria via culture is possible but not suitable for the diagnosis of acute leptospirosis due to the fastidious growth of the bacteria, which often requires specific culture media and might take several weeks to provide a positive result [9,10]. PCR-based strategies give faster results and demonstrate high sensitivity and specificity to detect *Leptospira* from urine, cerebrospinal fluid, or blood samples during the early stages of the disease [11] as well as in the urine, kidney, or genital tract of chronic animal carriers [12,13].

The environmental persistence of *Leptospira* and its epidemiology rely on the chronic renal colonisation of reservoir animals [14]. A reservoir may remain symptom-free while excreting the bacteria in its urine, either transiently or for its entire life [4,12]. Cattle are recognised as the maintenance host for serovar Hardjo (serogroup Sejroe), encompassing serovar Hardjobovis and Hardjoprajitno [5], and infection of cows with this serovar typically results in chronic infection and can lead to abortion, fertility disorders, and decrease in milk yield [4,5]. Therefore, the disease has an important economic impact due to both reproductive and non-reproductive losses to production [15]. Occurring at human-cattle-environment interfaces, bovine leptospirosis is considered an occupational zoonotic disease to e.g., farm workers and veterinarians [3] and represents a challenge for public and animal health.

Leptospirosis in cattle has been reported worldwide. Previous reviews referring to bovine leptospirosis, were conducted for Africa [16] and Latin America [17], however, to date, no work has summarised the knowledge on cattle leptospirosis from a European perspective, although essential to develop One Health strategies to prevent and manage outbreaks at human-cattle interfaces. To characterise the epidemiology of bovine leptospirosis in Europe, we conducted a systematic literature review and examined the extent and nature of the knowledge pertaining to this topic over a 20-year period, 2001-2021.

## 2 Methods

### 2.1 Literature search strategy

From 7 June to 26 August 2021, we performed a systematic literature search, using four electronic databases: Pubmed, Web of Science, Scopus, and CABI. The search queries included the following keywords “lepto*”, “cattle” “cows,” and “cow” (Supplementary material 1: Table 1). Additional papers were identified through internet-based search engines such as Google and Google Scholar and by hand-searches of the references cited in the reviewed studies. We considered articles published between 1 January 2001 and the date of search (26 August 2021), thereby covering over 20 years.

### 2.2 Paper selection and screening

First, citation data, title and abstracts were compiled and de-duplicated in Mendeley Reference Manager. Two reviewers (CS and ADL) independently screened all titles and abstracts. Titles/abstracts were selected if they contained qualitative and/or quantitative data on leptospirosis in cattle (e.g. data on clinical signs, prevalence, risk factors, circulating strains/serovars/serogroups) and if the study was conducted in a European country, as defined by the most common geographical definition of Europe, i.e. the land bordered by the Arctic Ocean to the north, the Atlantic Ocean to the west, the Mediterranean Sea to the south, and the Ural Mountains to the east. Case reports, outbreak description, epidemiologic surveys, reviews, or epidemiological reports were included.

Articles deemed eligible in the first round of screening were retrieved in full text format and reviewed independently by CS and ADL. Papers were excluded if the study was not pertaining to bovine leptospirosis, was performed outside Europe and/or was written in languages other than English, German, or French. Editorials, commentaries, book chapters, and conference proceedings were excluded. We also excluded papers dealing with immunology, vaccine strategy or efficacy, and diagnostic tools or methods. Disagreements between reviewers were discussed and resolved by consensus.

### 2.3 Data extraction and synthesis

Two reviewers (CS and ADL) extracted the following information from included papers: bibliographic information (including citation, type of paper, year of publication), study purpose and design, geographic location of the study, sampling unit (animal or herd), number of sampling unit(s) investigated, sample type(s), laboratory methods, positivity threshold, study period, reported prevalence and/or incidence, reported serogroup(s) and/or serovar(s) and/or genomospecies, clinical presentation, histological or necropsy findings, and production type (i.e. dairy, beef, mixed). For each serovar, when not provided, the serogroup was retrieved from the literature [18]. Moreover, we assessed the One Health-ness of each study by checking for the mention of the term “One Health” and examining whether each study explored compartments beyond cattle, such as human, environment, or other animals.

We also collected information on risk factors associated with *Leptospira* infection, i.e. investigated risk factor(s), assessed outcome(s), whether or not a statistical analysis was performed, relationships between the dependent variable and the risk factor (e.g. positive, negative, not evidenced), statistical model used, statistics reported and value, 95% confidence interval, and *p*-value. To obtain a more comprehensive understanding of the risk associated with *Leptospira* infection in European cattle, the risk factors were clustered into seven broader categories (Supplementary material 2: Appendix A). Finally, two additional independent reviewers (JS and JJ) performed a final curation and validation of the extracted data.

Summary statistics and figures were computed in R v.4.0.3 [19]. Figures were produced using the packages ggplot2 [20], ggalluvial [21], and forestplot [22].

## 3 Results

### 3.1 Selected studies

**Fig. 1:**
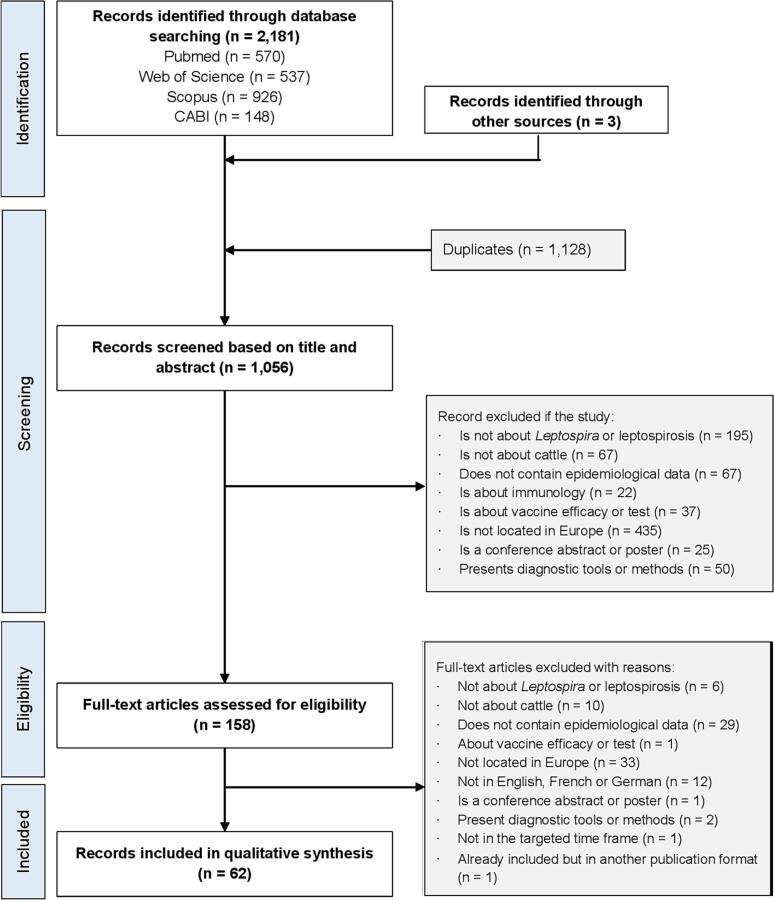
Review process flow chart.

Fig. 1 summarises the search strategy. The database search retrieved 2181 articles; after exclusion of duplicates (*n* = 1128), title/abstract of 1056 records were screened, of which, 158 were subjected to full-text screening. Many studies had to be excluded based on more than one criterion; numbers given in Fig. 1 indicate the primary exclusion criterion that was identified. A total of 62 articles were ultimately included in the systematic review. The details of the included studies are shown in Supplementary material 2: Appendix B.

The annual number of published papers on bovine leptospirosis showed a positive trend from 2001 to 2021 (Supplementary material 3: Fig. 1), with just over half of the studies published between 2014 and 2020 (33/62, 53.2%). Covering 18 European countries, the geographic distribution of the included studies exhibited spatial heterogeneity, with the highest number of studies conducted in the United Kingdom (*n* = 13, 21%) [[23], [24], [25], [26], [27], [28], [29], [30], [31], [32], [33], [34], [35]], the Republic of Ireland (*n* = 8, 12.9%) [[36], [37], [38], [39], [40], [41], [42], [43]], and France (*n* = 8, 12.9%) [[44], [45], [46], [47], [48], [49], [50], [51]] (Supplementary material 3: Fig. 2). Four papers described a case-control study (6.5%), 16 were clinical case investigations (25.8%), two were cohort studies (3.2%), 36 were cross-sectional studies (58.1%), and seven were longitudinal studies (11.3%) (three studies employed more than one research design).

Most of the studies examined dairy cattle (*n* = 23, 37.1%) [23,29,30,[32], [33], [34], [35],[37], [38], [39],^43^,^49^,^50^,[52], [53], [54], [55], [56], [57], [58], [59], [60], [61]] whereas beef cattle were occasionally considered (*n* = 7, 11.3%) [31,36,40,41,48,51,62]; four studies investigated both production types (4.8%) [45,[63], [64], [65]]. Nearly half of the studies did not mention the production type.

### 3.2 Laboratory methods

The MAT was the most employed method (*n* = 36 studies, 58.1%) and was predominantly performed on serum samples [42,[44], [45], [46], [47], [48], [49], [50], [51], [52], [53],[56], [57], [58], [59], [60], [61], [62], [63], [64],[66], [67], [68], [69], [70], [71], [72], [73], [74], [75], [76], [77], [78], [79], [80]], although one study used pleural fluids from aborted foetuses as an alternative [81]. The cut-off value (i.e. the threshold that defines whether a test result is positive or negative) used was highly variable, ranging between 1:5 and 1:1000 [42,[44], [45], [46], [47], [48],[51], [52], [53],^56^,[58], [59], [60], [61], [62], [63], [64],[67], [68], [69], [70], [71], [72], [73], [74], [75], [76], [77],^79^,^81^,^82^]. Similarly, the number of strains included in the MAT panel showed large disparities, from 1 to 24 strains (median = 10.5 strains per panel) [42,[44], [45], [46], [47],[50], [51], [52],^56^,^57^,^59^,^60^,[62], [63], [64],[66], [67], [68], [69], [70],[72], [73], [74], [75], [76], [77],^79^,^80^]. The ELISA was also widely used (*n* = 19, 30.6%) and generally performed to detect specific antibodies against serovar Hardjo, on serum [29,31,34,36,40,41,65,70,82,83] or milk samples [29,30,[32], [33], [34], [35],[37], [38], [39],^54^,^55^,^82^]. The agglutinin-absorption test (AAT) was used in one study [53].

PCR was occasionally performed (*n* = 11 studies, 17.7%) for the direct detection of *Leptospira*, e.g. in organs from aborted foetuses and stillborn calves, placenta, urine or blood from adult cows [48,51,57,59,60,62,64,70,73,81] as well as air samples [83]. Isolation of *Leptospira* by culture was rarely (*n* = 3 studies, 4.8%) and unsuccessfully attempted from urine, placental cotyledons, and water samples [48,62,65]. Immunofluorescence assays (*n* = 2) [28,73] and histology (*n* = 1) [73] were also marginally used in the studies. Similarly, genotyping methods were seldom employed, including multi-locus sequence typing (MLST, *n* = 2) [70,84], high-resolution melting analysis (HRMA) [81], and sequencing of the *lfb1* gene (*n* = 1) [81].

### 3.3 Occurrence of *Leptospira* infection

Epidemiological parameters (i.e. (sero)prevalence, incidence, or risk) were estimated at individual level (*n* = 44 studies) [[23], [24], [25], [26], [27], [28], [29],[36], [37], [38], [39],^42^,^44^,[48], [49], [50], [51],^53^,[56], [57], [58], [59], [60], [61], [62],[65], [66], [67], [68], [69], [70], [71], [72], [73], [74], [75], [76], [77], [78], [79], [80], [81], [82],^84^], herd level (*n* = 14) (i.e. a herd was defined as positive for leptospirosis if at least one animal within this herd was tested positive) [[30], [31], [32], [33],[36], [37], [38], [39], [40],^43^,^46^,^47^,^54^,^55^], or both (*n* = 8) [34,35,41,45,52,63,64,83]. The seroprevalence of leptospirosis in European cattle varied largely between countries and studies (Table 1). Overall, Belgium, France, Italy, Republic of Ireland, Spain, Ukraine, and the United Kingdom reported relatively high seroprevalences of leptospirosis in cattle whereas Bosnia and Herzegovina, Bulgaria, the Netherlands, and Sweden reported relatively low seroprevalences. Within countries, seroprevalence estimates exhibited great variations, e.g. in Belgium [60,65,70,71,81], France [44,45,[48], [49], [50], [51]], Spain [52,56,63], Ukraine [66,77,80] and the United Kingdom [[29], [30], [31], [32], [33], [34], [35]].

**Table 1:** Leptospira occurrence in cattle in Europe, 2001-2021. Seroprevalence: detection of antibodies through serological methods (ELISA, MAT, AAT), prevalence: detection of the presence of Leptospira by PCR, immunofluorescence assay, or culture. Seroprevalences and prevalences are given all laboratory methods combined, respectively.

Country	Epidemiological unit^a^	Seroprevalence range (No. of studies)	Prevalence range (No. of studies)	Type(s) of production system investigated (No. of studies)	References
Belgium	Animal	0% – 95% (5)	3.7% – 80.8% (2)	Dairy (1), dairy and beef (1), NA (3)	[60,65,70,71,81]
Bosnia and Herzegovina	Animal	1.6% (1)	NA	Dairy (1)	[61]
Bulgaria	Animal	0.4% (1)	NA	NA (1)	[79]
Croatia	Animal	1% – 22.6% (2)	NA	NA (2)	[75,78]
France	Animal	11.9% – 100% (6)	NA	Dairy (2), beef (2), dairy, beef and mixed (1), NA (1)	[44,45,[48], [49], [50], [51]]
France	Herd	38% – 100% (2)	NA	Dairy, beef and mixed (1), NA (1)	[45,46]
Germany	Animal	1.8% (1)	NA	Dairy and beef (1)	[64]
Germany	Herd	3.4% – 14.9% (1)	0.7% (1)	Dairy and beef (1)	[64]
Greece	Animal	12.6% (1)	NA	NA (1)	[68]
Italy	Animal	0.5% – 38.7% (4)	50% – 100% (1)	Beef (1), NA (3)	[62,67,69,74]
Netherlands	Herd	1.1% (1)	NA	Dairy (1)	[54]
Poland	Animal	0% – 26.8% (4)	0% (2)	Dairy (1), NA (3)	[57,72,76,83]
Poland	Herd	3.2% (1)	0% (1)	Dairy (1), NA (1)	[55,83]
Republic of Ireland	Animal	30.1% – 55.1% (2)	NA	Beef (1), NA (1)	[41,42]
Republic of Ireland	Herd	75.9% – 93.3% (5)	NA	Beef (3), Dairy (2)	[[36], [37], [38],^40^,^41^]
Slovakia	Animal	52 positive cattle (sample size unknown)	NA	Dairy (1)	[53]
Spain	Animal	6.4% – 43.3% (3)	NA	Dairy (2), dairy and beef (1)	[52,56,63]
Spain	Herd	36.2% – 100% (2)	NA	Dairy (1), Dairy and beef (1)	[52,63]
Sweden	Animal	0% – 0.98% (2)	NA	Dairy (1), NA (1)	[58,82]
Switzerland	Animal	21.4% (1)	1.8% – 12.5% (2)	Dairy (1), NA (1)	[59,73]
Ukraine	Animal	0.19% – 64.6% (3)	NA	NA (3)	[66,77,80]
United Kingdom	Animal	13.7% – 41.7% (3)	4.3% – 100% (6)	Dairy (4), NA (6)	[[23], [24], [25], [26], [27], [28], [29],^34^,^35^]
United Kingdom	Herd	21.9% – 80% (6)	NA	Dairy (5), beef (1)	[[30], [31], [32], [33], [34], [35]]

a Epidemiological unit investigated, e.g. if herd, the prevalence gives the number of herds positive divided by the total number of herds investigated (no information on the individual animals).

### 3.4 Diversity and geographic distribution of *Leptospira* serogroups, and genomospecies in cattle in Europe

**Fig. 2:**
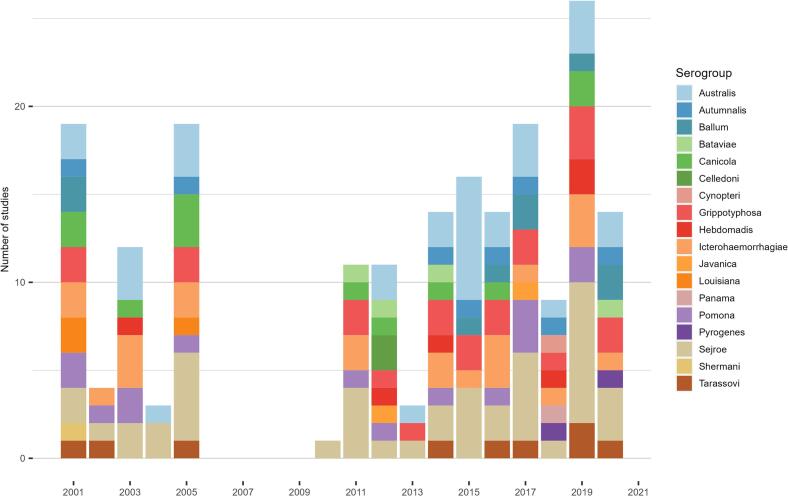
Circulating *Leptospira* serogroups in European cattle (determined by MAT) reported in the included papers, 2001-2021. X-axis represents years of publication.

Overall, 53 studies reported 18 different circulating serogroups, in cattle in Europe during the period 2001-2021 (Table 2). The five most reported *Leptospira* serogroups (identified by MAT) were Sejroe (*n* = 31 studies), Australis (*n* = 22), Grippotyphosa (*n* = 22), Icterohaemorrhagiae (*n* = 20), and Pomona (*n* = 14). The highest serogroup diversity was found in Western Europe (for detailed data on serogroups, see Supplementary material 2: Appendix B). The serogroup diversity was relatively stable over the 20 years (Fig. 2). Three serogroups were sporadically reported, namely Cynopteri and Panama in 2018 only [70] and Pyrogenes in 2018 [70] and 2020 [81], even though these serogroups were tested in other studies [44,46,48,51,53,60,66,72,76].

**Table 2:** Serovars and serogroups reported in cattle in Europe, 2001-2021. We used a regionalisation of Europe as provided by UNStats (https://unstats.un.org/unsd/methodology/m49/): Results are provided at serogroup or serovar level, depending on the information available in the study. For each serovar, when not provided, the serogroup was retrieved from the literature [18].

Serogroup	Serovar	Eastern Europe^a^ [53,55,57,66,72,76,77,79,80]	Northern Europe^b^ [23,25,[29], [30], [31], [32], [33], [34], [35], [36], [37], [38], [39], [40], [41], [42], [43],^58^]	Southern Europe^c^ [52,56,[61], [62], [63],[67], [68], [69],^74^,^75^,^84^]	Western Europe^d^ [44,[46], [47], [48], [49], [50], [51],^54^,^59^,^60^,^64^,^70^,^71^,^73^,^81^]
Australis	Australis	X		X	X
Australis	Bratislava	X		X	X
Australis	Muenchen				X
Australis	NA	X			X
Autumnalis	Autumnalis			X	X
Autumnalis	NA				X
Ballum	Ballum			X	X
Ballum	Castellonis			X	
Ballum	NA				X
Bataviae	Bataviae	X		X	
Bataviae	NA				X
Canicola	Canicola	X		X	X
Canicola	NA	X			
Celledoni	Celledoni	X			
Celledoni	Cynopteri	X			
Cynopteri	NA				X
Grippotyphosa	Grippotyphosa	X		X	X
Grippotyphosa	NA	X		X	X
Hebdomadis	Hebdomadis	X		X	
Hebdomadis	NA	X			X
Icterohaemorrhagiae	Copenhageni			X	X
Icterohaemorrhagiae	Icterohaemorrhagiae	X		X	X
Icterohaemorrhagiae	NA	X			X
Javanica	Poi	X			
Javanica	NA				X
Louisiana	Louisiana			X	
Panama	NA				X
Pomona	Mazdok			X	
Pomona	Pomona	X		X	X
Pomona	NA	X		X	
Pyrogenes	NA				X
Sejroe	Hardjo	X	X	X	X
Sejroe	Istrica	X			
Sejroe	Polonica	X			
Sejroe	Saxkoebing	X		X	X
Sejroe	Sejroe	X			X
Sejroe	Strain mouse 2A (domestic strain)		X		
Sejroe	Wolffi				X
Sejroe	NA	X		X	X
Shermani	Shermani			X	
Tarassovi	Tarassovi	X		X	X
Tarassovi	NA	X			X

a Eastern Europe: Bulgaria, Poland, Slovakia, Ukraine.

b Northern Europe: Republic of Ireland, Sweden, United Kingdom.

c Southern Europe: Bosnia and Herzegovina, Croatia, Greece, Italy, Portugal, Spain.

d Western Europe: Belgium, France, Germany, Switzerland, Netherlands.

Three genomospecies were characterised in European cattle over the study period: *Leptospira interrogans* in Belgium (serogroup Icterohaemorrhagiae and Australis) [70,81], Portugal (serogroup Pomona) [84], and Republic of Ireland (serogroup Sejroe) [43], *Leptospira kirschneri* (serogroup Grippotyphosa) in Belgium [70,81]. and *Leptospira borgpetersenii* (serogroup Sejroe) in Portugal [84].

### 3.5 Clinical signs and histo-pathological findings associated with bovine leptospirosis in Europe

Twenty-nine studies (46.8%), from 13 different countries, described clinical signs and/or histo-pathological findings associated with *Leptospira* infection in cattle. Clinical signs of leptospirosis differed largely with respect to the age of the animal, with acute forms of the disease typically affecting calves and foetuses, while chronic forms are generally observed in adults (Table 3). We also evidenced that clinical signs depicted in dairy versus beef cattle were slightly different (Supplementary material 3: Fig. 3).

**Table 3:** Clinical manifestations associated with bovine leptospirosis in Europe, 2001-2021. The table presents the number of studies reporting each sign and the age category of the animals, as specified in the study (*n* = 16 studies).

Clinical sign	Number of studies	Adult	Calf	Aborted foetus	Age not specified	References
Abortion	17	X			X	[[23], [24], [25], [26],^48^,^49^,^51^,^52^,^59^,^62^,^64^,^70^,^71^,^73^,^78^,^79^,^81^]
Fertility disorders	7	X			X	[43,47,48,52,56,60,62]
Subclinical	4	X			X	[50,[53], [54], [55]]
Icterus	4		X	X		[28,51,70,71]
Perinatal mortality	4		X		X	[24,26,43,57]
Death	3		X		X	[27,49,51]
Decrease in milk production	3				X	[29,49,50]
Photosensitisation	3				X	[[49], [50], [51]]
Hyperthermia	3	X			X	[49,50,62]
Apathy	2	X			X	[49,62]
Diarrhoea	2		X		X	[28,49]
Haematuria	2				X	[50,51]
Absence of sucking reflex	1		X			[51]
Anisocytosis	1		X			[51]
Bilateral epistaxis	1		X			[51]
Cough	1				X	[49]
Embryonic mortality	1				X	[49]
Haemorrhagic diarrhoea	1				X	[49]
Haemolactation	1				X	[49]
Hepatonephritis	1				X	[51]
Hypothermia	1		X			[51]
Interdigital ulcers	1				X	[49]
Meconium retention	1		X			[51]
Normochromic normocytic anaemia	1		X			[51]
Oedema of the eyelids	1				X	[49]
Permanent decubitus	1		X			[51]
Petechiae	1		X			[51]
Platelet macrocytosis	1		X			[51]
Recumbency	1		X			[28]
Stomatitis	1				X	[49]
Swollen pasterns	1				X	[49]
Tachycardia	1		X			[51]

Abortion was the most frequently reported sign of *Leptospira* infection in cattle (17/29, 58.6%) [[23], [24], [25], [26],^48^,^49^,^51^,^52^,^59^,^62^,^64^,^70^,^71^,^73^,^78^,^79^,^81^], followed by fertility disorders (7/29, 24.1%), including suboptimal reproductive performances such as prolonged calving intervals and poor conception rates [43,47,48,52,56,60,62]. These symptoms have been generally associated with chronic infections [47,48,50,54,55,60], however, Grippi et al. [62] mentioned infertility and abortion, associated with sudden death, in an acute episode of bovine leptospirosis. Manifestations of an acute infection included a sudden drop in milk production [29,49,50], hyperthermia [49,50,62], haemoglobinuria [50,51], and icterus [28,51,71]. In severe cases, death [27,49,51] and perinatal mortality [24,26,28,43,57] were described. Clinical signs may be aggravated by concomitant infections, e.g. with *Anaplasma phagocytophilum* and bovine viral diarrhoea (BVD) virus [49]. Other signs, e.g. photosensitisation [[49], [50], [51]], diarrhoea [28,49], haematuria [50,51], bilateral epistaxis [51], cough [51], swollen pasterns [49], interdigital ulcers [49], oedema of the eyelids [49] or abnormal blood counts [51] were sporadically described. Subclinical leptospirosis was reported in four studies [50,[53], [54], [55]].

Pathological findings were mostly described in calves and included pathological kidney alterations [27,28,51,81], icterus [27,51,81], congested liver or hepatomegaly [27,28,51], splenomegaly [51,71,81] and petechiae on the lungs and heart [51]. Histopathological changes affected mostly the liver, spleen, and kidneys [27,28,51,71,81]. Placentitis, necrosis, mononuclear or mixed inflammatory cell infiltrates, vasculitis and the presence of intracytoplasmatic and extracellular bacteria were observed on adult carcasses [73].

### 3.6 Risk factors for bovine leptospirosis in Europe

Risk factors for bovine leptospirosis were addressed in 28 studies (45.2%) [29,30,32,33,[36], [37], [38], [39], [40], [41],[48], [49], [50],^52^,[54], [55], [56],^58^,^60^,^61^,^63^,^64^,^71^,^72^,^75^,^77^,^80^,^81^], of which, 18 used a statistical approach to quantify the association between the risk factor(s) and *Leptospira* infection [30,32,33,[36], [37], [38], [39], [40], [41],^52^,^55^,^56^,^58^,^61^,^63^,^71^,^72^,^81^] whereas the others evaluated the risk qualitatively through field observations [29,[48], [49], [50],^54^,^55^,^60^,^64^,^75^,^77^,^80^] (Supplementary material 2: Appendix C). Overall, we found that 53 risk factors and 17 dependent variables were investigated (Supplementary material 1: Table 2). Thirty-one risk factors were studied in dairy cattle versus 17 in beef herds, showing preferential investigations of dairy production systems.

**Fig. 3:**
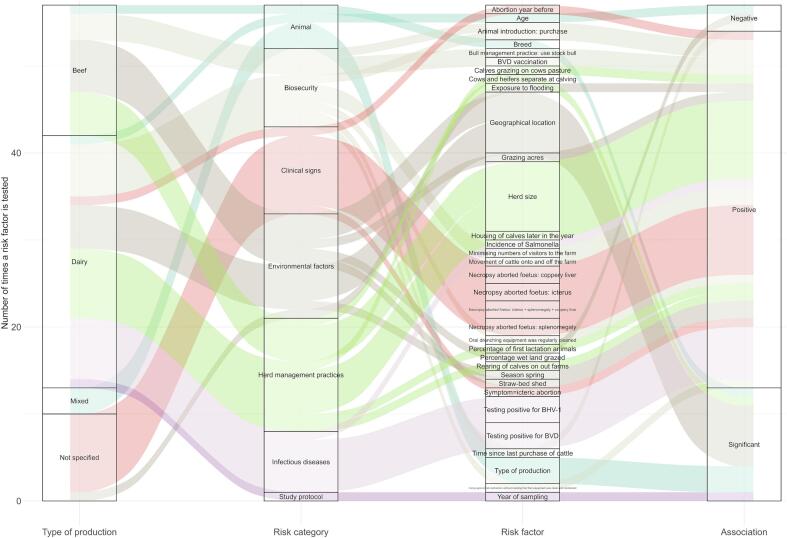
Sankey diagram showing the type of production, the risk factor categories, the risk factors investigated, and the direction of the association between risk factors and *Leptospira*-infection in cattle, Europe, 2001-2021. Only studies that have performed a statistical analysis (*n* = 18) are included and only significant risk factors are displayed. Colours represent the risk categories. The y-axis represents the number of times a risk factor was tested across the 18 studies; x-axes represent the production type, risk category, and statistical association between the risk factor and the presence of *Leptospira*. Association may be positive or negative; “significant” means that the association is statistically significant, but no direction is provided.

The main risk factors statistically and positively associated with *Leptospira* infection in cattle included: i) environmental factors, such as the geographic location of the animal (or herd) [33,37,40,41,61], exposure to flooding [72], the season spring [56], and access to pasture [40]; ii) herd management practices, such as the herd size [30,32,36,37,[39], [40], [41]] (although some studies found no statistical relationship [33,55,63]), rearing calves off farm, co-grazing of calves and cows, housing the calves later in the year [39], segregating heifers and cows at calving [40], and the percentage of primiparous cows in the herd [39]; iii) factors related to biosecurity, such as the employment of agricultural contractors untrained on biosecurity [39], the purchase of animals [32,36,54] (although two studies did not confirm this result [30,40]), the movement of cattle onto and off the farm [39], and the use of a stock bull [40]: iv) comorbidity with infectious diseases (e.g. past or co-occurring infection with BVD, bovine herpes virus 1 (BHV-1), *Salmonella*) [30,32,33,38]; v) clinical conditions, such as a recent history of abortion [52] (especially icteric abortion [71,81]); and vi) individual factors, such as age and sex (which showed inconsistent effects on the risk of infection by *Leptospira* [41,58,60]), breed [41], or type of production [63], with dairy herds being significantly more at risk of infection with *Leptospira* serovar Copenhageni, Grippotyphosa, and Tarassovi than beef herds [63]. Finally, factors related to the study design (e.g. date of sampling) may have an impact on the seroprevalence of *Leptospira* [61] (although, date of sampling might be a confounding factor reflecting the influence of e.g. the season, weather, or specific conditions at time of sampling) (Fig. 3, Supplementary material 3: Fig. 4).

In contrast, minimising the number of visitors in farms [39] or increasing the percentage of wetland grazed [40] were shown to decrease significantly the probability of infection. Interestingly, a previous *Leptospira* infection was not reported as a risk factor for subsequent infection [40]. Finally, although not tested statistically, the presence of rodents [48,50], the use of pig manure for the cow grazing pasture [60], extreme weather conditions [75], and access to natural water sources [29,60] were also noted as risk factors of infection in cattle. Surprisingly, herds where cleaning of drenching equipment was performed were more likely to test positive for antibodies to *Leptospira* serovar Hardjo [39], as were cattle vaccinated against the BVD virus [36]. One possible reason for these associations is that the cleaning of drenching equipment and vaccination might have been operated in response to the presence of the bacteria or other infectious diseases in the herd [39].

### 3.7 One Health-ness of the selected papers

Only one paper [47] explicitly mentioned the term “One Health”, and none of the studies examined all the traditional One Health aspects (animal, human, environment). Six papers (9.7%) incorporated data or samples from both cattle and humans, underscoring the zoonotic nature of the disease [53,67,69,74,75,82]. The environmental aspect was investigated in two studies (3.2%) [65,83] whereas other animal species (such as pigs, sheep, and dogs) were examined in 21 studies (33.9%) [[23], [24], [25], [26], [27],^44^,^47^,^48^,^60^,[66], [67], [68], [69],^72^,[74], [75], [76],^79^,[82], [83], [84]], showcasing a broader perspective beyond cattle.

## 4 Discussion

Despite the apparent growing risk, the impact on cattle and human health, and the potential severe economic losses related to bovine leptospirosis, this review shows that there is a lack of comprehensive data on *Leptospira* infection in cattle in the European region, with studies published from 18 European countries only. To some extent, the geographic distribution of the publications included in this review reflects the distribution of the bovine population in Europe, with a large proportion of studies conducted in the United Kingdom, Ireland, and France.

The occurrence of leptospirosis in European cattle exhibited significant variation, both between and within countries. This variability may be influenced by several factors, including the geographical scale of the study (national, regional, or at farm level), geographic location, and study design (e.g. case investigation versus retrospective study, sample size, laboratory methods). Knowledge of the (sero)prevalence of bovine leptospirosis in a geographic region is essential for veterinary practitioners, but also medical doctors, to include or exclude the disease in their differential diagnoses and reduce under- and misdiagnoses. However, the prevalence of the bacteria in cattle in Europe remains largely elusive since many studies involved a relatively small number of animals while clinical case reports represented one quarter of the included studies.

This review demonstrates that MAT is the prevailing technique used to confirm leptospirosis in cattle across Europe, despite significant heterogeneity in laboratory procedures. Notably, the MAT cut-off values displayed significant variations, indicating a lack of consensus over which titre should be used for a positive result. In addition, the MAT antigen panel was found to be extremely variable between studies, which prevents accurately mapping serogroup distribution in Europe. Furthermore, intrinsic limitations of the method, i.e. possible cross-reactivity between serogroups, does not allow the identification of the infecting serogroup with absolute certitude [85] and generally, the agglutinating serogroup with the highest MAT titres is the only one reported. These drawbacks narrow our understanding of the epidemiology of the circulating serogroups.

To provide comparable data at European level, harmonised protocols to investigate, diagnose, and report cases of leptospirosis in cattle are necessary. For example, cattle-specific cut-off values for the MAT should be defined. Likewise, a pan-European consensus is needed regarding the minimum panel of serogroups to be included in the MAT, which could further be regionally optimised with locally isolated strains [86], as recommended by, the World Organisation for Animal Health (WOAH) [85]. Harmonised protocols could further help identifying possible associations between infecting serogroups and clinical signs in cattle.

This review demonstrates that the serogroups Sejroe, Australis, Grippotyphosa, Icterohaemorrhagiae, and Pomona are the most reported ones in cattle in Europe. Yet, those serogroups were the most used in the MAT panels across reviewed studies, which may bias the overall epidemiological picture. Nevertheless, this finding supports previous observations, e.g. from South America [17] Africa [16], Malaysia [87], and New Zealand [88]. These serogroups have also been associated with human cases globally [16,89,90], stressing the need for further research on the transmission potential of the bacteria at human-cattle-environment interfaces, that would address the disease through a One Health lens.

As described in tropical and sub-tropical areas [91,92], New Zealand [93], and Australia [94], the most recognised and reported clinical signs of bovine leptospirosis in European cattle are abortions and fertility disorders. Therefore, in case of abortion events in European cattle, leptospirosis should be considered as a differential diagnosis among other, more classical, infectious causes of abortion, i.e. *Brucella* spp., *Neospora caninum*, *Coxiella burnetii*, BVD virus, BHV-1, or *Salmonella enterica* serotype Dublin [95]. However, clinical signs can vary widely, making a field diagnosis challenging and emphasising the importance to rely on standardised laboratory tests.

This review provides a comprehensive overview of the risk factors of *Leptospira* infection in cattle in Europe despite the heterogeneity of the study designs, which challenges the comparison of the results. The most important risk factors of bovine leptospirosis were related to biosecurity measures and the environment. Local or even regional conditions influence the presence of *Leptospira* in the environment as well as host-bacteria interactions. Several factors related to the soil and water pH, temperature, or composition of the environmental microbiome may determine the possibility of persistence of *Leptospira* outside its animal host [96]. Climatic conditions in Europe are becoming increasingly suitable for the survival and transmission of water- and rodent-borne diseases, including leptospirosis [97]. Extreme weather events compounding the impact of changes in land use (especially urbanisation) intensify the direct and indirect contacts between leptospires, humans, and animal hosts [89], therefore increasing the risk for public and animal health. Overall, this review evidences the multiplicity and complexity of the risk factors associated with *Leptospira* exposure in cattle. These findings should motivate local and national health authorities, veterinarians, and farmers to implement integrated disease prevention and control measures at farm or regional level.

Research on cattle leptospirosis in Europe suffers several data and research gaps. Remarkably, we did not find any paper reporting a successful isolation of a *Leptospira* strain from naturally-infected cattle in Europe between 2001 and 2021. Although the recovery of *Leptospira* from field samples is extremely challenging, the isolation of local strains from infected animals, humans, or the environment is essential to optimise the MAT panel for the serological diagnosis of leptospirosis in humans and animals [85,98] but also to delineate relevant One Health interventions [99]. We highlighted a lack of molecular data on *Leptospira* strains circulating in cattle. The isolation and molecular characterisation of *Leptospira* from cattle and their environment would advance knowledge on the epidemiology, ecology, and pathogenesis of the bacteria, and would have practical applications in the prevention, surveillance, and control of the disease in both animals and humans. More efforts are needed in this direction. This review also stresses a lack of large-scale studies, necessary for drawing representative conclusions and achieving sufficient statistical power, and points out that dairy herds are disproportionally more frequently investigated compared to beef herds, leading to a data gap regarding the clinical signs, prevalence, and impact of the disease in beef cattle. We also demonstrated that the zoonotic and One Health aspect of bovine leptospirosis is largely neglected in Europe, highlighting a potential gap in understanding and addressing the epidemiological link between cattle, human, and the broader ecological context. Additionally, we noted a lack of data on the risk related to artificial insemination and a limited investigation of rodents as a source of *Leptospira* infection in cattle herds. Finally, in the last 20 years, no study investigated bovine genital leptospirosis (BGL) in Europe, although studies from Brazil have evidenced a relatively high prevalence of the disease [13] and point toward the recognition of BGL as a distinct syndrome [6].

## 5 Conclusions

Research on bovine leptospirosis is generally under-resourced while the disease is globally neglected [100], including in Europe, where a limited number of countries have investigated and reported the disease. Considering the veterinary and public health importance of leptospirosis as well as its economic impact, it is crucial to raise awareness among stakeholders, including farmers, veterinarians, and other health professionals, in areas where the disease is not (yet) endemic. This is especially important in Europe where this zoonotic disease is (re-)emerging in humans, but also in animals. Moreover, intensification of livestock farming in certain regions of Europe, concomitant with an increasing trend toward herd grazing outdoors in other areas will also certainly play a major role in the future regional incidences of leptospirosis in cattle, probably increasing regional contrasts. Local studies are essential to advance our understanding of the epidemiology of bovine leptospirosis and therefore develop and implement relevant, locally-adapted prevention and control strategies. Nevertheless, an overview of the epidemiology of the disease at continental scale, as presented here, can yield novel insights into its epidemiological features in a One Health context.

## Funding

This study was funded by the federal state of Lower Austria (Land Niederösterreich) (WST3-F-5033836/001-2020). The funders had no role in study design, data collection and analysis, decision to publish, or preparation of the manuscript.

Declarations of interest: none.

## Author contributions

**Cynthia Sohm:** Data curation, Formal analysis, Investigation, Software, Validation, Visualization, Writing – Original draft preparation, Writing – Review and Editing.

**Janina Steiner:** Data curation, Formal analysis, Investigation, Writing – Original draft preparation, Writing – Review and Editing.

**Julia Jöbstl:** Data curation, Investigation, Writing – Original draft preparation, Writing – Review and Editing.

**Thomas Wittek:** Funding acquisition, Writing – Review and Editing.

**Clair Firth:** Funding acquisition, Writing – Review and Editing.

**Romana Steinparzer:** Funding acquisition, Project administration, Writing – Review and Editing.

**Amélie Desvars-Larrive:** Conceptualization, Data curation, Formal analysis, Funding acquisition, Investigation, Methodology, Project administration, Resources, Software, Supervision, Validation, Visualization, Writing – Original draft preparation, Writing – Review and Editing.

## Declaration of Competing Interest

The authors declare that they have no known competing financial interests or personal relationships that could have appeared to influence the work reported in this paper.

## Data Availability

All relevant data are within the manuscript and its Supporting Information files.

## Appendix A Supplementary data

Supplementary material 1 Supplementary tables. Supplementary material 1

Supplementary material 2 Extracted data on cattle leptospirosis retrieved from the 62 reviewed papers, Europe, 2001-2021. Appendix A: Risk categories attributed to the different risk factors; Appendix B: List of selected papers and extracted data (except risk factors); Appendix C: Extracted data on risk factors of leptospirosis in European cattle; Appendix D: Metadata. Supplementary material 2
