# Supplementary material for: A systematic review on leptospirosis in cattle: A European perspective
Source: One Health. 2023 Jul 27;17:100608. doi: 10.1016/j.onehlt.2023.100608 (PMC10416059; doi:10.1016/j.onehlt.2023.100608)
Supplement: Supplementary material 1 — Supplementary tables. [file mmc1.pdf]

Supplementary material 1

Table 1. Search queries used in the different databases for this systematic literature review.

Database	Search query
Pubmed	(((lepto*) AND ((cattle) OR (cow) OR (cows))
Scopus	TITLE-ABS-KEY (lepto*) AND TITLE-ABS-KEY (cattle OR cow OR cows)
Web of Science	(((AB=(lepto*)) AND (AB=(cattle) OR AB=(cow) OR AB=(cows))))
CABI	((lepto*) AND ((cattle) OR (cow) OR (cows)) AND yr:[2001 TO 2021]) AND (((geographic-location:(("Italy" OR "Poland" OR "Irish Republic" OR "Germany" OR "Spain" OR "Russia" OR "Wales" OR "Netherlands" OR "Lombardy" OR "Austria" OR "Bulgaria" OR "UK" OR "England" OR "Croatia" OR "Nordic Countries" OR "Czech Republic" OR "Bosnia-Hercegovina" OR "Belgium" OR "Switzerland" OR "Northern Ireland" OR "Slovakia" OR "Europe" OR "France"))) (language:(("English" OR "German" OR "French")))))

Table 2. Dependent variables used in the studies investigating risk factors of cattle leptospirosis in Europe, 2001-2021 (n = 28 studies).

Dependent variable	Number of studies	References
Herd (sero)positivity to *Leptospira*	9	[30,32,33,37-40,64,72]
Within-herd seroprevalence	7	[30,32,33,36,41,55,63]
Seroprevalence	6	[41,58,61,75,77,80]
Animal (sero)positivity to *Leptospira*	5	[33,48,50,60,71]
Incidence of *Leptospira*	2	[38,54]
Animal seropositivity to *Leptospira* (sv. Bratislava)	1	[52]
Herd antibody titre level	1	[72]
Herd seroprevalence	1	[41]
Incidence of *Leptospira* sv. Grippotyphosa	1	[56]
Incidence of *Leptospira* sg. Australis (sv. Bratislava)	1	[56]
PCR positive in aborted foetus	1	[81]
Seropositivity to *Leptospira* in aborted dam (titre 1:100)	1	[81]
Severity of clinical symptoms	1	[49]
Within-herd antibody titre level	1	[37]
Within-herd seroprevalence (sv. Copenhageni)	1	[63]
Within-herd seroprevalence (sv. Grippotyphosa)	1	[63]
Within-herd seroprevalence (sv Tarassovi)	1	[63]

sv.: serovar; sg.: serogroup.
