# Supplementary material for: A systematic review on leptospirosis in cattle: A European perspective
Source: One Health. 2023 Jul 27;17:100608. doi: 10.1016/j.onehlt.2023.100608 (PMC10416059; doi:10.1016/j.onehlt.2023.100608)

Supplementary material 3

Figure 1. Bar chart of the distribution of the year of publication of the 62 studies included in the systematic review on cattle leptospirosis in Europe, 2001-2021.

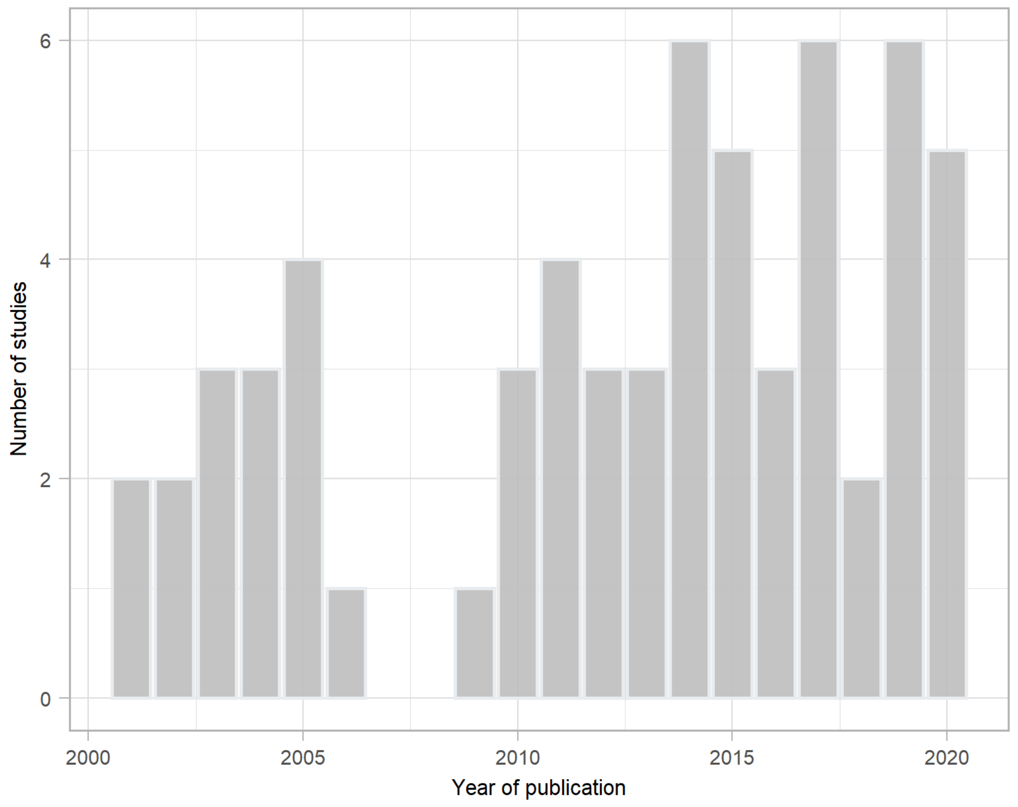

Figure 2. Number of studies per country pertaining to bovine leptospirosis published in Europe between 2001 and 2021 and selected for this systematic review. Grey: no studies included. The map was produced using the R package "rnaturalearth" from South, A. (2017). rnaturalearth: World Map Data from Natural Earth, R package version 0.1.0 (https://CRAN.R-project.org/package=rnaturalearth) and is license-free.

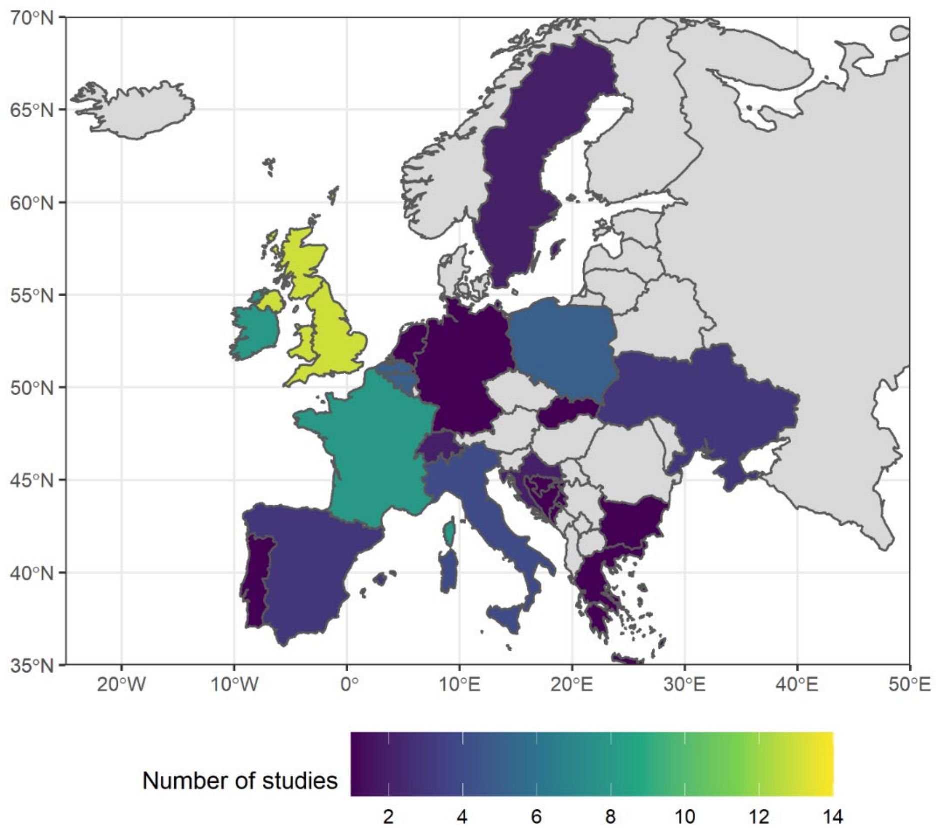

Figure 3. Cleveland dot plot displaying the number of studies reporting at least one clinical sign associated with bovine leptospirosis in Europe per type of production, 2001-2021.

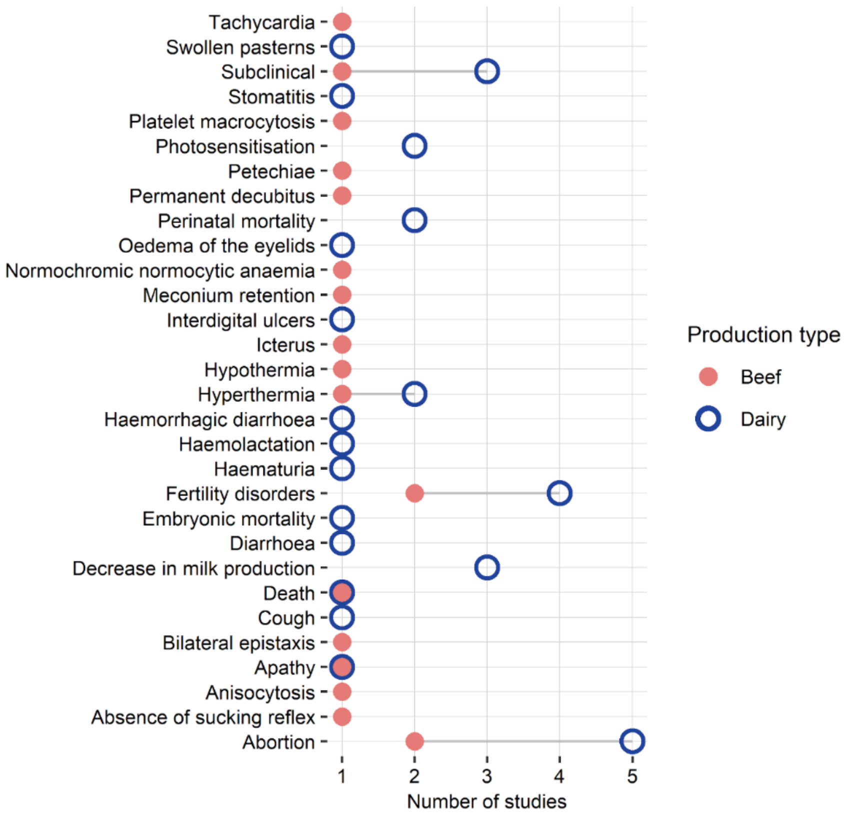

Figure 4. Forest plot of the risk factors of bovine leptospirosis, Europe, 2001-2021. The figure presents the odds ratios (OR) and 95% confidence interval (95% CI) for nine European studies that used this metrics to evaluate risk factors of leptospirosis in cattle. Note that if 95% CI contains the value 1, the OR is not significant.

Studies	Dependent variable	Risk factor	OR
Atxaerandio et al., 2005	Animal (sero)positivity to Leptospira (serovar Bratislava)	Abortion year before	3.23
Bishop et al., 2010	Herd (sero)positivity to Leptospira	Animal introduction: purchase	16.3
Williams et al., 2014	Herd (sero)positivity to Leptospira	Animal introduction: purchase	2.57
Bishop et al., 2010	Within-herd seroprevalence	Bull management practice: hiring bulls	3.2
Ryan et al., 2012b	Herd (sero)positivity to Leptospira	Bull management practice: use stock bull	3.056
O' Doherty et al., 2014	Herd (sero)positivity to Leptospira	Calves grazing on cows pasture	13.69
Ryan et al., 2012b	Herd (sero)positivity to Leptospira	Geographical location	1.378
Ryan et al., 2012b	Herd (sero)positivity to Leptospira	Grazing acres	1.607
Velasova et al., 2017	Within-herd seroprevalence	Herd size	1
O' Doherty et al., 2014	Herd (sero)positivity to Leptospira	Herd size	0.02
Ryan et al., 2012b	Herd (sero)positivity to Leptospira	Herd size	2.039
Ryan et al., 2012b	Herd (sero)positivity to Leptospira	Herd size	2.039
O' Doherty et al., 2014	Herd (sero)positivity to Leptospira	Movement of cattle onto and off the farm	15.15
Grégoire et al., 2020	Seropositivity to Leptospira in aborted dam (titre 1:100)	Necropsy aborted foetus: coppery liver	4.43
Grégoire et al., 2020	PCR positive in aborted foetus	Necropsy aborted foetus: coppery liver	7.6
Grégoire et al., 2020	Seropositivity to Leptospira in aborted dam (titre 1:100)	Necropsy aborted foetus: extended haemorrhagic pattern	3.49
Grégoire et al., 2020	PCR positive in aborted foetus	Necropsy aborted foetus: extended haemorrhagic pattern	0.58
Grégoire et al., 2020	Seropositivity to Leptospira in aborted dam (titre 1:100)	Necropsy aborted foetus: icterus	13.55
Grégoire et al., 2020	PCR positive in aborted foetus	Necropsy aborted foetus: icterus	70.91
Grégoire et al., 2020	Seropositivity to Leptospira in aborted dam (titre 1:100)	Necropsy aborted foetus: icterus + splenomegaly + coppery liver	6.44
Grégoire et al., 2020	PCR positive in aborted foetus	Necropsy aborted foetus: icterus + splenomegaly + coppery liver	9
Grégoire et al., 2020	Seropositivity to Leptospira in aborted dam (titre 1:100)	Necropsy aborted foetus: peri-renal haemorrhages	0.75
Grégoire et al., 2020	PCR positive in aborted foetus	Necropsy aborted foetus: peri-renal haemorrhages	1.22
Grégoire et al., 2020	Seropositivity to Leptospira in aborted dam (titre 1:100)	Necropsy aborted foetus: splenomegaly	5.69
Grégoire et al., 2020	PCR positive in aborted foetus	Necropsy aborted foetus: splenomegaly	19.03
O' Doherty et al., 2014	Herd (sero)positivity to Leptospira	Oral drenching equipment was regularly cleaned	0.02
Ryan et al., 2012b	Herd (sero)positivity to Leptospira	Percentage wet land grazed	0.588
Guitián et al., 2001	Incidence of Leptospira serogroup Australis (Bratislava)	Season fall	0.63
Guitián et al., 2001	Incidence of Leptospira Grippotyphosa	Season fall	0.33
Guitián et al., 2001	Incidence of Leptospira serogroup Australis (Bratislava)	Season spring	0.38
Guitián et al., 2001	Incidence of Leptospira Grippotyphosa	Season spring	3.74
Guitián et al., 2001	Incidence of Leptospira serogroup Australis (Bratislava)	Season summer	1.16
Guitián et al., 2001	Incidence of Leptospira Grippotyphosa	Season summer	1.36
Ryan et al., 2012b	Herd (sero)positivity to Leptospira	Straw-bed shed	4.304
Delooz et al., 2015	Animal (sero)positivity to Leptospira	Symptom=icteric abortion	48
Bishop et al., 2010	Herd (sero)positivity to Leptospira	Testing positive for BHV-1	7.5
Bishop et al., 2010	Herd (sero)positivity to Leptospira	Testing positive for BVD	42

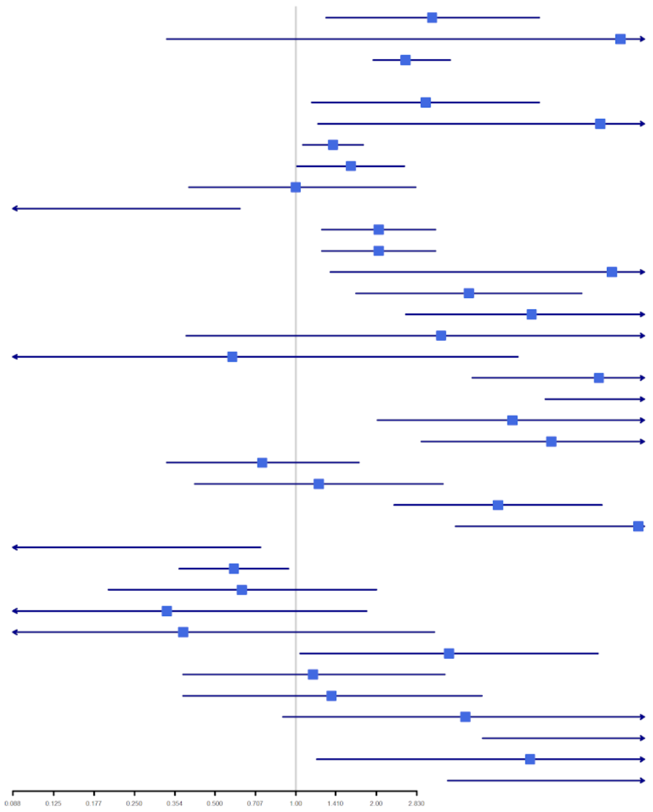

Supplement: Supplementary material 3 — Supplementary figures. [file mmc3.pdf]
